# Neural Network-Based Decoding Input Stimulus Data Based on Recurrent Neural Network Neural Activity Pattern

**DOI:** 10.1134/S001249662201001X

**Published:** 2022-03-17

**Authors:** S. I. Bartsev, P. M. Baturina, G. M. Markova

**Affiliations:** 1 Institute of Biophysics, Siberian Branch, Russian Academy of Sciences, 660036 Krasnoyarsk, Russia; 2 Siberian Federal University, 660041 Krasnoyarsk, Russia

**Keywords:** delayed match-to-sample test, neural activity, dynamic coding, classification of neural activity patterns

## Abstract

The paper reports the assessment of the possibility to recover information obtained using an artificial neural network via inspecting neural activity patterns. A simple recurrent neural network forms dynamic excitation patterns for storing data on input stimulus in the course of the advanced delayed match to sample test with varying duration of pause between the received stimuli. Information stored in these patterns can be used by the neural network at any moment within the specified interval (three to six clock cycles), whereby it appears possible to detect invariant representation of received stimulus. To identify these representations, the neural network-based decoding method that shows 100% efficiency of received stimuli recognition has been suggested. This method allows for identification the minimum subset of neurons, the excitation pattern of which contains comprehensive information about the stimulus received by the neural network.

The possibility of reconstructing the content of data processed by the brain from the dynamic patterns of neural activity is the key task in the Neural Correlates of Consciousness (NCC) concept [1]. According to the current views and based on neurophysiological data [2–4], encoding task-relevant information in working memory is very dynamic since it is represented by widely varying patterns of neuronal activity.

It is known that coding the information about the external stimulus received by the recurrent artificial neural network (RNN) in the course of a delayed matching-to-sample (DMS) test is also a dynamic process [5]. Because in the present study the pause length between the acquisition of two stimuli was fixed, the only requirement was to reach the desired point in the RNN neural activity space by the time the second stimulus arrives [2]. If the pause length between the first and second stimuli is chosen randomly from a given interval, then the problem of how the information available for use at any moment during the pause can be stored in the RNN becomes much more challenging.

The aim of the present work was to assess the possibility of identifying the stimulus received by RNN based on the neural activity pattern in the period when the network stores data about the stimulus in its working memory in the ready-to-respond state. The task which requires RNN storing information in the form of a neuronal activity pattern for a certain period of time is a DMS test.

Simple RNNs with two inputs and 25 internal neurons were used. The neuron number was determined empirically as the minimum number required to complete the task. In contrast to 20-neuron RNNs, the 25-neuron ones could be successfully trained (to the error as low as about 10^–5^). Verification showed that 30-neuron RNNs were easier to train to pass the DMS test. However, 25-neuron RNNs are more convenient in terms of analysis, while focus on using the minimum possible neuron set is in line with the neural correlates approach.

The initial values for weight coefficients were chosen randomly from within the (–0.025; 0.025) range. RNN response $y_{o}^{{(t)}}$ at the time point *t* was recorded at the two output neurons:

1 $$y_{h}^{{(t)}} = {{f}_{h}}({{W}_{h}}y_{h}^{{(t - 1)}} + {{W}_{i}}{{x}^{{(t)}}}),\quad y_{o}^{{(t)}} = {{f}_{o}}({{W}_{o}}y_{h}^{{(t)}}),$$

where *W*_*h*_, *W*_*i*_, *W*_*o*_ are the weight coefficient matrices for the internal neurons, input, and output neurons, respectively; *x*^(*t*)^ is the vector of input signals at the time point *t*; $y_{h}^{{(t)}}$ and $y_{h}^{{(t - 1)}}$ are vectors describing internal neuron excitation levels at the time points *t* and *t* – 1. The *f*_*h*_(.) and  *f*_*o*_(.) functions are activation functions for internal and output neurons, respectively. For the sake of simplicity, neuron displacements are omitted from the equations.

The activation function for internal neurons was sigmoidal (2a). The piecewise linear activation function (2b) for the output neurons was used to obtain an accurate 0/1 output signal.

2 $$\begin{gathered} a)\;{{f}_{h}}(x) = \frac{1}{2}\left( {\frac{x}{{a + \,\,{\text{|}}x{\text{|}}}} + 1} \right){\text{,}} \\ b)\;{{f}_{o}}(x) = \left\{ \begin{gathered} 0,\quad {\text{if}}\;\;x \leqslant 0, \hfill \\ b \cdot x,\quad {\text{if}}\;\;x > 0\;\& \;x < 1, \hfill \\ 1,\quad {\text{if}}\;\;x \geqslant 1. \hfill \\ \end{gathered} \right. \\ \end{gathered} $$

The parameters of the activation functions (2) had the values *a* = 0.1 and *b* = 1, which were selected empirically for the fastest RNN training. The synapse modification step was set equal to 10^–3^.

The RNN was trained using the error backpropagation algorithm. Since the structure of the trained network does not depend on the training algorithm [6, 7], its specific form is not important for the analysis of its functioning. The quadratic loss function was used:

3 $$C = \frac{1}{2}\sum\limits_{i = 1}^N {{{{(y_{i}^{{(t)}} - \delta _{i}^{{(t)}})}}^{2}}} ,$$

where $y_{i}^{{(t)}}$ and $\delta _{i}^{{(t)}}$ the present and the required signals at the *i*th RNN output neuron at the time point *t* and *N* is the output neuron number.

RNN could receive one of the three input stimuli: *A*, (01); *B*, (10); and *C*, (11). Given that (00) is the absence of any stimuli, the full set of possible stimuli for a given number of inputs was used. The DMS test was conducted as follows. One of the randomly chosen stimuli (*A*, *B*, *C*) arrived to the RNN input at random time points. The stimulus was presented to the RNN as a single beat. Then a pause 3 to 6 beats long followed during which no signal arrived at the RNN input. The length of the pause was also determined randomly. Then a second stimulus was presented once, also chosen at random. The third beat after the second stimulus was the RNN response (10) or (01) which depended on whether the two acquired stimuli were the same or different. Then, after a relaxation period of at least 9 beats, the next training cycle began. Thus, the training sample was continuously generated during the training process, which allowed us to neglect the probability that significant fragments of the input stream of quasi-random events would be repeated.

The trained RNNs were subjected to the DMS test in the function mode in the same continuous quasi-random event stream ensuring non-reproducibility of the test signal sample.

To identify the stimulus received by the RNN, we used data on the network’s neural activity during the pause between the first and second stimuli. During this period from the 3 to 6 beats after receiving the first stimulus, the RNN stored the information about this stimulus in the form of a neural activity pattern. The neural activity dynamics revealed high variability of excitation patterns in the interval between the stimuli with no clear signs of statics. To identify the stimulus in the function mode, the pause between the stimuli was set to be of maximum length (6 beats).

As a control, the centroid method [8] was used to identify the dynamic invariant of RNN neural activity during the information storage period. The activity of the RNN neurons at each moment of time was represented as a point in the multidimensional neuronal activity space with *R*^*N*^ dimensions, where N is the number of neurons in the RNN. By averaging the activity at four consecutive beats during the information storage period, the most likely location of the points corresponding to each of the three possible stimuli was calculated:

4 $$\overline E _{t}^{\alpha } = \frac{1}{4}\sum\limits_{t = 3}^6 {E_{{t,n}}^{\alpha }} ,$$

where $E_{{t,n}}^{\alpha }$ is the activity at the RNN neuron *n* at the time point *t* after receiving the stimulus α(*A*, *B*, *C*). In this case, activity values from the training sample were used. The three points thus obtained were the *A*, *B*, and *C* centroids, respectively. To identify the stimulus, squared Euclidean distances from each centroid to the points from the test sample were calculated:

5 $$D_{t}^{\alpha } = \sum\limits_{t = 1}^N {(\overline E _{n}^{\alpha } - E_{{t,n}}^{{}}} {{)}^{2}},$$

where ${{E}_{{t,n}}}$ is the activity at the RNN neuron *n* at the time point *t* obtained from the test sample.

Identification of the stimulus, information about which was stored encoded in the RNN neural activity was carried out according to which of the three centroids was closest to the point in question:

6 $${\text{Sti}}{{{\text{m}}}_{t}} = \left\{ \begin{gathered} A,\quad {\text{if}}\;\;\min (D_{t}^{A},D_{t}^{B},D_{t}^{C}) = D_{t}^{A}, \hfill \\ B,\quad {\text{if}}\;\;\min (D_{t}^{A},D_{t}^{B},D_{t}^{C}) = D_{t}^{B}, \hfill \\ C,\quad {\text{if}}\;\;\min (D_{t}^{A},D_{t}^{B},D_{t}^{C}) = D_{t}^{C}. \hfill \\ \end{gathered} \right.$$

The resulting stimulus type $Sti{{m}_{t}}$ was compared with the real one available for each test data set and based on this the accuracy of identification was evaluated.

Although in a number of cases the centroid method allowed correct identification of stimulus based on the neural activity pattern, its efficiency did not exceed 80%, which could be explained by the high signal variability (Fig. 1). At different points within the stimulus storage period, the accuracy of identification using this method was different.

**Fig. 1.**
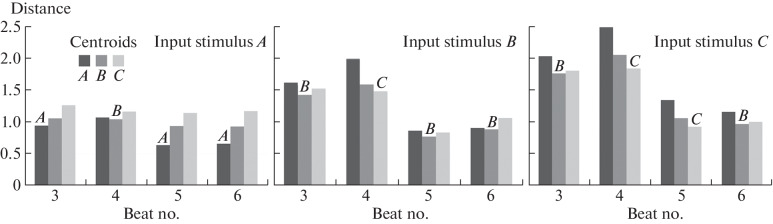
Using the centroid method to identify RNN-derived stimuli. Columns, distances from the points in the neuronal activity space to each of the three centroids. The type of the input stimulus received by the RNN is indicated at the top of the diagram. Letters above each group of columns indicate the type of the stimulus identified in the corresponding beat based on the minimum distance to the centroids.

As the next step, the invariant representation storing the information about the received stimulus during four beats was extracted with the aid of an additional neural network, a neural network decoder (DN) (Fig. 2).

**Fig. 2.**
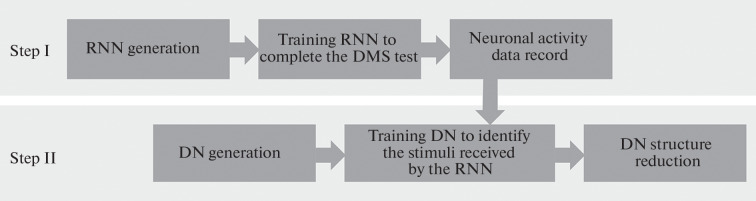
DN training experiment outline.

A single-layer neural network consisting of three neurons with linear response was used as the DN (2b). Each neuron had a modifiable synapse with each of the inputs, the number of which was equal to the number of neurons in the RNN. The DN produced 1 at one of the three neurons corresponding to the assigned stimulus and zeros at the others. The error backpropagation algorithm was used for training. The loss function was quadratic as it was in the previous case (3).

The input data for the DN were the neural activity of a particular RNN which was subjected to the DMS test. The neural activity of the RNN was recorded line by line. The line contained the activity of each of the 25 RNN neurons at a given moment in time (3, 4, 5, or 6 beats after the first stimulus), and the stimulus, the information about which was stored in the RNN at that time, was associated with it. For all the trained RNNs, 72 lines were recorded, which were distributed between the training and test samples randomly.

An individual DN needed to be trained for each trained RNN, indicating the uniqueness of the neural network’s internal stimulus representation. The trained DNs decoded the stimuli represented by RNNs with 100% accuracy. The DN structure was further reduced, namely, the synapses with the lowest absolute values were sequentially equated to zero, and at each step, the DN was trained again until it reached its original performance level. This procedure was stopped when DN performance started to decline. As a result, a group of six or seven neurons was selected for each of the trained RNNs, whose activity was used to decode the received stimuli.

The neural activity invariants corresponding to the conditions for recognizing each of the three stimuli may be localized in the multidimensional space of dynamic patterns. Towards this end, the sets of randomly generated numbers imitating the activities of RNN neurons selected for decoding were applied to the inputs of trained DNs. Those sets of random numbers which were identified as corresponding to any of the stimuli by the DN were selected and considered as points in the neural activity space representing the code of a certain stimulus.

Let us consider the structure of a particular DN as an example. This DN identified the six neurons from the original network numbered 2, 3, 8, 17, 20, 21 as significant and sufficient.

When the input data pass through the DN, the result of the calculation has the general form $w_{2}^{\alpha }{{x}_{2}} + w_{3}^{\alpha }{{x}_{3}} + w_{8}^{\alpha }{{x}_{8}}$ + $w_{{17}}^{\alpha }{{x}_{{17}}} + w_{{20}}^{\alpha }{{x}_{{20}}} + w_{{21}}^{\alpha }{{x}_{{21}}} = {{s}^{\alpha }}$, where $w_{i}^{\alpha }$ is a nonzero weight DN coefficient obtained after DN structure was reduced was reduced which links the *i* DN input with the neuron responsible for the recognition of the α stimulus, ${{x}_{i}}$ is the activity of the neuron *i* in the original RNN, and $\alpha = A,B,C$. When ${{s}^{A}} \geqslant 1,$ ${{s}^{B}} \leqslant 0,$ and ${{s}^{C}} \leqslant 0$, the DN identifies the resulting data set as storing the information about the stimulus *A*. The datasets for the stimuli *B* and *C* are identified in the same way.

Linear polynomials which allow identifying the invariants for the RNN considered as an example are as follows:

$$\begin{gathered} 0.158{{x}_{2}} - 0.142{{x}_{3}} + 0.461{{x}_{8}} \\ \, + 0.595{{x}_{{17}}} - 0.582{{x}_{{20}}} + 0.245{{x}_{{21}}} = {{s}^{A}}, \\ \end{gathered} $$

$$\begin{gathered} - 0.244{{x}_{2}} + 0.545{{x}_{3}} - 0.079{{x}_{8}} \\ \, - \,\,0.4{{x}_{{17}}} + 0.072{{x}_{{20}}} - 0.509{{x}_{{21}}} = {{s}^{B}}, \\ \end{gathered} $$

$$\begin{gathered} - 0.243{{x}_{2}} - 0.292{{x}_{3}} - 0.827{{x}_{8}} \\ \, - \,\,0.349{{x}_{{17}}} + 0.328{{x}_{{20}}} + 0.887{{x}_{{21}}} = {{s}^{C}}. \\ \end{gathered} $$

Principal component analysis showed that the points corresponding to invariant recognition property in the neuronal activity space form three compact clusters (see Fig. 3 for an example). Mapping the neuron activity onto the two-dimensional plane formed by the first and the second principal components is sufficient for recognition. The activity of one neuron among the six RNN neurons make insignificant contribution to the second principal component. This suggests that the activities of five neurons are sufficient for stimulus recognition for a given RNN, although such variant was not detected when reducing the DN structure. Therefore, principal component analysis or similar methods may be useful to finally minimize the number of stimulus coding invariant representations in the neural network.

**Fig. 3.**
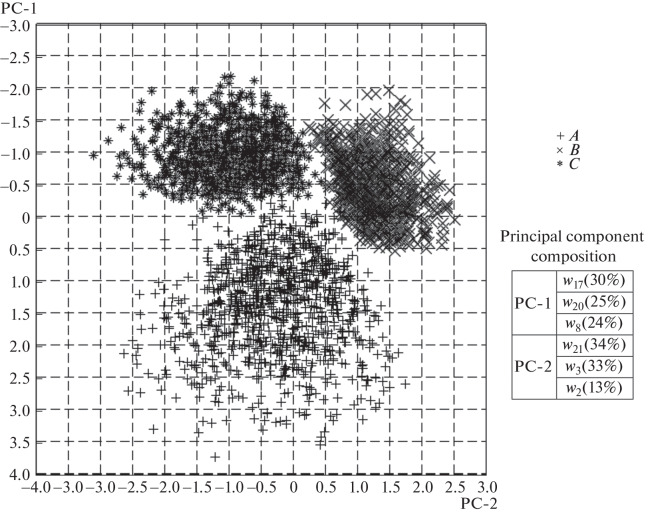
Invariant configurations corresponding to stimulus recognition conditions after the principal component analysis.

Based on the results obtained in the present work, we may conclude that despite the dynamic nature of neuronal activity enabling the storage of information about the received stimuli the stimulus type may be identified based on neuronal activity patterns. Neural network–based decoding method proposed in the present work allows to identify the dynamic neuronal activity invariant which represents a given stimulus with 100% accuracy. In addition, this approach implies the identification of the minimum set of neurons and, consequently, the minimum neuronal activity required to solve the task set for the neural network; hence, this approach is in line with the concept of neural correlates [1].
